# Vanadium removal and recovery from bauxite residue leachates by ion exchange

**DOI:** 10.1007/s11356-016-7514-3

**Published:** 2016-09-01

**Authors:** Helena I. Gomes, Ashley Jones, Mike Rogerson, Ian T. Burke, William M. Mayes

**Affiliations:** 1 School of Biological, Biomedical and Environmental Sciences, University of Hull, Cottingham Road, Hull, HU6 7RX UK; 2 Department of Geography, Environment and Earth Sciences, University of Hull, Cottingham Road, Hull, HU6 7RX UK; 3 School of Earth and Environment, University of Leeds, Leeds, LS2 9JT UK

**Keywords:** Red mud, Metal recovery, Alkaline drainage, Recycling, Sorption, Anion exchange resin

## Abstract

Bauxite residue is an important by-product of the alumina industry, and current management practices do not allow their full valorisation, especially with regard to the recovery of critical metals. This work aims to test the efficiency of ion exchange resins for vanadium (V) removal and recovery from bauxite residue leachates at alkaline pH (11.5 and 13). As an environmental pollutant, removal of V from leachates may be an obligation of bauxite residue disposal areas (BRDA) long-term management requirements. Vanadium removal from the leachate can be coupled with the recovery, and potentially can be used to offset long-term legacy treatment costs in legacy sites. Kinetics studies were performed to understand the adsorption process. The rate kinetics for the V adsorption was consistent with the pseudo-first-order kinetic model, with a higher adsorption rate for pH 11.5 (1.2 min^−1^). Adsorption isotherm data fitted better to Freundlich equations than to the Langmuir model. The maximum adsorption capacity (Langmuir value *q*_max_) was greatest for pH 13 (9.8 mg V g^−1^ resin). In column tests, breakthrough was reached at 70 bed volumes with the red mud leachate at pH 13, while no breakthrough was achieved with the effluent at pH 11.5. In regeneration, 42 and 76 % of V were eluted from the resin with 2 M NaOH from the red mud leachate at pH 13 and 11.5, respectively. Further optimization will be needed to upscale the treatment.

## Introduction

Bauxite residue or red mud is a major by-product of the aluminium industry, with an annual global production of 150 million t (Evans 2016) and a total inventory of 2.7 billion t (Binnemans et al. 2013). The current best practice in the industry is disposal in engineered bauxite residue disposal areas or BRDA, increasingly using dry stacking methods with equipment such as Amphirols to aid dewatering of the mud in order to compact and consolidate the residue, and treatment of any leachates that are produced (Evans 2016; Power et al. 2011). Partial neutralization using seawater, carbonation using waste carbon dioxide from ammonia production and accelerated carbonation using intensive farming methods are also used as best management practises (Evans 2016). The leachates generated by bauxite residue are enriched in a range of metal and metalloid oxyanions, namely As, Cr, Mo, Ni and Ga, and can have vanadium (V) concentrations in the range of 1.2–15.6 mg L^−1^ (Burke et al. 2013; Czop et al. 2011; Mayes et al. 2011). Vanadium is typically mobile in bauxite residue leachate, as it is present in its pentavalent form (Burke et al. 2012, 2013), which is toxic and a possible human carcinogen (IARC 2006). The treatment of these alkaline leachates by conventional methods (acid dosing and active aeration) is expensive, especially if it is to be continued for many decades after site closure (Evans 2015). Furthermore, buffering of bauxite residue leachate does not efficiently remove vanadium from solution (Burke et al. 2013) due to the mobility of vanadate from hyperalkaline conditions through to *circum* neutral pH (Takeno 2005). As such, there is a key need to develop remedial tools for V removal from alkaline leachates. Where this removal can be coupled with options for the recovery of vanadium and other critical metals from these hyperalkaline leachates (pH 9–13), there may be considerable scope for offsetting long-term legacy treatment costs.

Different materials have been used for vanadium removal from waters and wastewaters. Adsorbents such as activated carbon (Sharififard and Soleimani 2015), high-area C-cloth electrodes (Afkhami and Conway 2002) and metal oxide adsorbents (Naeem et al. 2007) have been used at bench scale for vanadium removal. The current research on removal of vanadium from waters and wastewaters shows growing trends towards the development of new adsorbent materials, such as nanoscale iron oxide-hydroxide-impregnated activated carbon (Sharififard et al. 2016), bisphosphonate nanocellulose (Sirviö et al. 2016), chitosan films (Cadaval et al. 2016) and flakes (Padilla-Rodríguez et al. 2015). Simultaneously, the use of waste materials as absorbents is also being studied (Hu et al. 2014; Hua et al. 2015; Leiviskä et al. 2015).

Vanadium has a medium risk of supply shortage and a high political risk (Hunt and Kraus 2013). It is also considered critical for the development of green technologies (Naden 2013) and renewable energy production and storage (Viebahn et al. 2015). The largest producers are China, Russia and South Africa (USGS 2015). Vanadium is mostly used in ferrous and non-ferrous alloys (Song et al. 2014), as well as for catalysts in automobiles, and in the chemical and polymer industries (Keränen et al. 2013; Navarro et al. 2007). The current recovery methods from secondary sources are based on ion exchange (Huang et al. 2010; Keränen et al. 2013; Li et al. 2013; Nguyen and Lee 2013; Zeng et al. 2009; Zhao et al. 2010), solvent extraction (Barik et al. 2014; Kim et al. 2014) and bioleaching (Mirazimi et al. 2015). Although, ion exchange resins have been used previously for V recovery or removal, limited data exist on feed solutions with a pH higher than 10 (Huang et al. 2010), and none with bauxite residue leachate.

The aim of this study was to investigate the efficacy of a basic anion exchange resin for the removal and recovery of vanadium from synthetic bauxite residue leachate solutions. The impact of pH and different vanadium concentrations were tested in batch tests. Experiments with columns allowed assessing breakthrough in removal and the feasibility of recovering vanadium.

## Materials and methods

### Chemicals

Two different bauxite residue leachates were generated in the laboratory and tested—an operating BRDA leachate (pH 13) and a post-closure BRDA effluent (pH 11.5) (Gräfe et al. 2011). Both were made with bauxite residue recovered from the dyke breach of the Ajka (Hungary) impoundment (Lat 47° 05′ 20 N, Long 14° 29′ 43 E) in December 2010 (Burke et al. 2012). The samples were kept refrigerated and, before use, dried at 60 °C for 48 h, and disaggregated using a mortar and pestle. The bauxite residue had carbonated after collection, so to fabricate the operating BRDA leachate, it was mixed with 0.1 M NaOH in a liquid to solid (L/S) ratio of 20 for 24 h. Before the experiments, the leachate was vacuum-filtered (0.2 μm). The post-closure BRDA effluent resulted from the dilution of the operating BRDA leachate with deionized water (15 MΩ cm ELGA Purelab water) and then enriched with divanadium pentoxide (general purpose grade, Fisher Chemical) to get a V concentration of approximately 5 mg L^−1^ (Clune et al. 2015; Higgins et al. 2015). Table 1 shows the average composition of the synthetic bauxite residue leachates used in this study, which shows ratios of major ion and trace element enrichment similar to environmental samples from Ajka leachate albeit at slightly lower concentrations.

**Table 1:** Average composition of the operating and post-closure synthetic bauxite residue leachate and comparison with the concentrations of major and selected trace elements found in Ajka (mg L^−1^), Hungary (Mayes et al. 2011)

	Synthetic operating BRDA leachate (*n* = 3)	Synthetic post-closure BRDA effluent (*n* = 3)	K1 sample, Ajka (01/12/2010)
pH	13.1 ± 0.1	11.5 ± 0.1	13.06
mg L^−1^			
Ca	1.02 ± 0.43	0.5 ± 0.4	1.3
Mg	<0.04	0.02 ± 0.01	0.001
K	10.3 ± 6.4	1.2 ± 0.9	85
Na	>1000	186.0 ± 139.0	701
Al	69 ± 14	2.0 ± 1.2	659
Si	2.4 ± 3.9	5.3 ± 4.6	0.7
P	0.3 ± 0.2	0.7 ± 0.02	–
S	19.9 ± 15.8	1.5 ± 1.1	–
As	1.08 ± 0.41	0.03 ± 0.02	3.6
Ga	0.1 ± 0.05	0.02 ± 0.01	2.3
Mo	0.3 ± 0.2	<0.007	4.1
V	4.6 ± 1.8	5.3 ± 0.1	5.7
W	0.2 ± 0.04	<0.007	0.5

Table 2 presents the properties of the resin used in the experiments: Amberlite®IRA-400 chloride form (Sigma-Aldrich). Prior to use, the resin was washed with water until the supernatant became clear and colourless, and then soaked in deionized water for 6 h, with 5 wt.% NaOH solution for 4 h, and again washed with deionized water until the supernatant pH was in the range of 8–9 (Huang et al. 2010). The resin was consequently used in the hydroxide form.

**Table 2:** Characteristics of Amberlite® IRA-400 (Mustafa et al. 2010)

Properties	
Polymer matrix	Polystyrene divinylbenzene copolymer
Functional group	–N^+^R_3_ (quaternary ammonium)
Physical form	Pale yellow translucent beads
Particle size	600–750 μm
Ionic form	Cl^−^
Exchange capacity	2.6–3 eq kg ^−1^ (dry mass)
Effective size	0.3–0.9 mm
Operating temperature	80 °C (maximum)
pH range	0–14

Batch tests containing 10 g of wet resin in 150 mL of solution were conducted at room temperature (20 ± 1 °C) in conical flasks (250 mL) to investigate the rate of V removal from solution. The flasks were mixed using a magnetic stirrer at 150 rpm. Aqueous samples (10 mL) were taken after 1, 2, 3, 4, 5, 10, 15, 20, 25 and 30 min. To establish the adsorption isotherms, further batch tests were made with 20 mL of the bauxite residue leachates and 1, 5, 10, 20 and 50 g L^−1^ of the ion exchange resin, stirred at 150 rpm for 30 min in an orbital shaker.

### Column experiments

Column experiments were conducted in ϕ1.8 × 35 cm acrylic (Plexiglas) tubes. Hydroxyl conditioned resin was wet-packed into the column. The resin bed was backwashed with 200 mL of deionized water, allowed to stand for 20 min, and then flushed with deionized water, before introduction the bauxite residue leachate enriched with V_2_O_5_ at a flow-rate of 6 mL min^−1^ (see Table 3 for details). The bauxite residue leachate was produced as described in “Chemicals” section. Samples of the column effluent were collected every 30 min and analysed to determine vanadium concentrations. After finishing the stock solution, the column was flushed with 200 mL of deionized water, and then eluted with 2 M NaOH (200 mL) at a flow rate of 6 mL min^−1^. The eluent was collected in 10 mL fractions.

**Table 3:** Experimental parameters of the column experiments

Parameters	Test 1	Test 2
Feed solution	Operating BRDA leachate	Post-closure BRDA effluent
Feed grade target ion—vanadium (mg L^−1^)	3	5
Flow rate (mL min^−1^)	6	6
Flow direction	Downstream	Downstream
Resin volume (mL)	5	5
Bed volume—BV (L)	0.051	0.051
Bed depth (cm)	2	2
Flow rate (BV h^−1^)	72	72
Flow velocity (cm^−1^ s^−1^)	0.033	0.033
Duration (h)	5	5

### Analyses

All aqueous samples (10 mL) were filtered (0.45 μm, MCE Membrane Millex HA) and preserved with HNO_3_ (Romil SpA™ Super Purity Acid). The analyses were made using a Varian inductively coupled plasma optical emission spectrometer (ICP-OES). A blank and standard suite were analysed every ten samples to check instrument calibration, and wavelengths were selected by standard methods (USEPA Method 200.7). The calibration curve was prepared with 1, 5 and 10 mg L^−1^ standards. The quality control was made with a certified reference material (CRM-ES AT-1529, lot number 1319108).

### Data analysis

Hydrochemical data was analysed using the software PHREEQC Interactive v.3.1.7.9213 with the MINTEQ V4 database to determine the speciation of the most important elements and the stability of the solutions.

## Results

### Effect of contact time

**Fig. 1:**
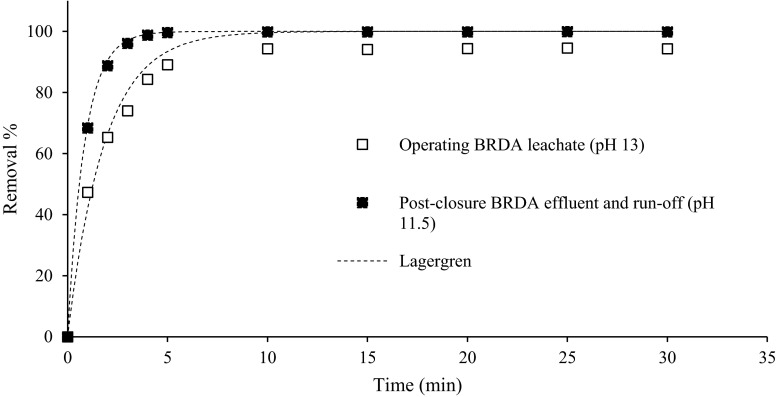
Removal of vanadium in the ion exchange resin in time with operating and post-closure bauxite residue leachate (pH 13 and 11.5) and the pseudo-first-order kinetics curves (Lagergren)

Figure 1 shows the effect of contact time on the V removal in batch tests with the bauxite residue leachate (pH 13 and 11.5). There was a rapid initial adsorption of V, and both leachates reached a steady state within the 30-min test period. The bauxite residue leachate showed a fast removal in the first 5 min; equilibrium was reached immediately after. The pH 11.5 leachate showed the highest removal percentages (99.9 %) while the pH 13 leachate only reached 94 %.

The kinetics of V adsorption on the resin was studied from the time versus percent removal curves. The rate of adsorption was analysed using the model proposed by Lagergren (Eq. 1) (Hua et al. 2015):

1 $$ \log \left({q}_e-{q}_t\right)\kern0.5em =\kern0.5em  \log {q}_e-\frac{k_1}{2.303}t $$

where *k*_1_ (min^−1^) is the adsorption rate constant, *q*_t_ is the amount adsorbed at time *t* (min) and *q*_e_ denotes the amount adsorbed at equilibrium, both in mg g^−1^. The rate constants (*k*_1_) for the tested conditions were calculated from the linear least square method and are given in Table 4 along with the correlation coefficient (*r* ^2^). The best fits were obtained for pH 11.5 leachate (Table 4), with the adsorption rate being more than the double that of the pH 13 leachate.

**Table 4:** Pseudo-first-order rate constants and coefficient of determination (*r* ^2^) for V removal

Feed solution	Initial pH	Final pH	Initial [V] (mg L^−1^)	Pseudo-first-order kinetics (Lagergren)	
				*k*_1_ (min^−1^)	*r* ^2^
Post-closure effluent	11.5	11.6	5.3	1.181	0.997
Operating BRDA leachate	13.3	13.2	4.7	0.545	0.985

### Adsorption isotherms

The experimental data for V adsorption to the resin were fitted with Langmuir and Freundlich adsorption isotherms. The Langmuir isotherm assumes monolayer adsorption onto a surface with a finite number of identical sites, homogeneous distribution of sorption sites and sorption energies, without interactions between the sorbed molecules, and is described by:

2 $$ {q}_e=\frac{b\ {q}_{\max }{C}_e}{1\kern0.5em +\kern0.5em b{C}_e} $$

where *q*_e_ and *C*_e_ are equilibrium concentrations of V in the adsorbed (mg g^−1^) and liquid phases (mg L^−1^), respectively. The Langmuir constants are the maximum adsorption capacity *q*_max_ and *b* energy of adsorption.

On the other hand, Freundlich adsorption isotherm describes the adsorption equilibrium onto a heterogeneous surface with uniform energy (non-ideal adsorption), and is expressed by:

3 $$ {q}_e={K}_f{C}_e^{\raisebox{1ex}{$1$}\!\left/ \!\raisebox{-1ex}{$n$}\right.} $$

where *q*_e_ and *C*_e_ are the equilibrium concentrations of metal in the adsorbed (mg g^−1^) and liquid phases (mg L^−1^), respectively. *K*_f_ and *n* are the Freundlich constants that are related to adsorption capacity and intensity, respectively.

**Fig. 2:**
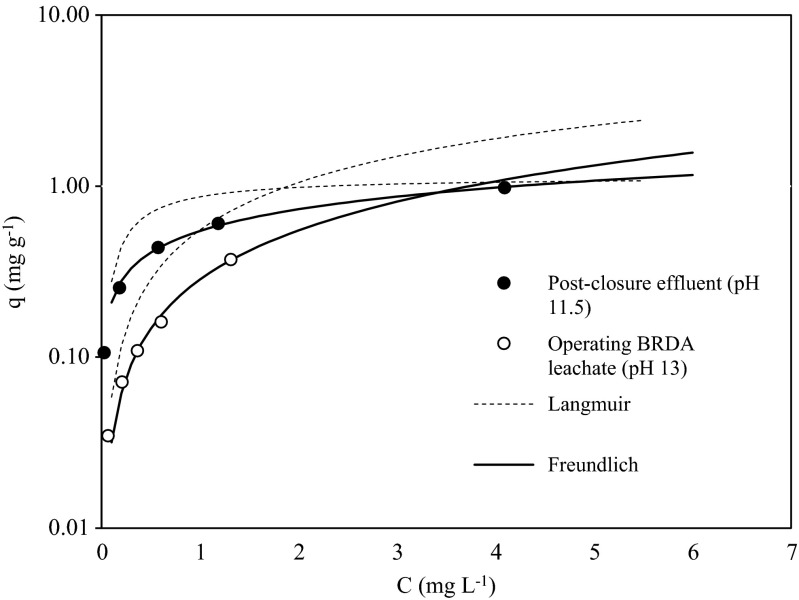
Isotherm curves and experimental data for the operating BDRA neat residue leachate (pH 13) and the post-closure effluent and runoff (pH 11.5)

Table 5 and Fig. 2 show the Langmuir and Freundlich isotherms fitting to the experimental data. The Freundlich isotherms fit the data better (*r* ^2^ > 0.99). The values of the Freundlich constant, 1/n were less than 1, which revealed that the exchange process is favourable. The Freundlich adsorption capacity constant *K*_f_ at pH 11.5 is almost double than the one at pH 13.0. Maximum adsorption capacity values (*q*_max_) are higher at pH 13 (10 mg V g^−1^ resin).

**Table 5:** Langmuir and Freundlich isotherm constants and coefficient of determination (*R* ^2^) for adsorption of V in the bauxite residue leachates

	pH	Langmuir isotherm			Freundlich isotherm		
		*q*_max_ (mg g^−1^)	*b* (L mg^−1^)	*R* ^2^	*K*_f_	*n*	*R* ^2^
Post-closure effluent	11.5	1.135	3.218	0.859	0.547	2.383	0.999
Operating BRDA leachate	13.3	9.759	0.060	0.919	0.285	1.050	0.993

### Competing ions: removal efficiency

**Fig. 3:**
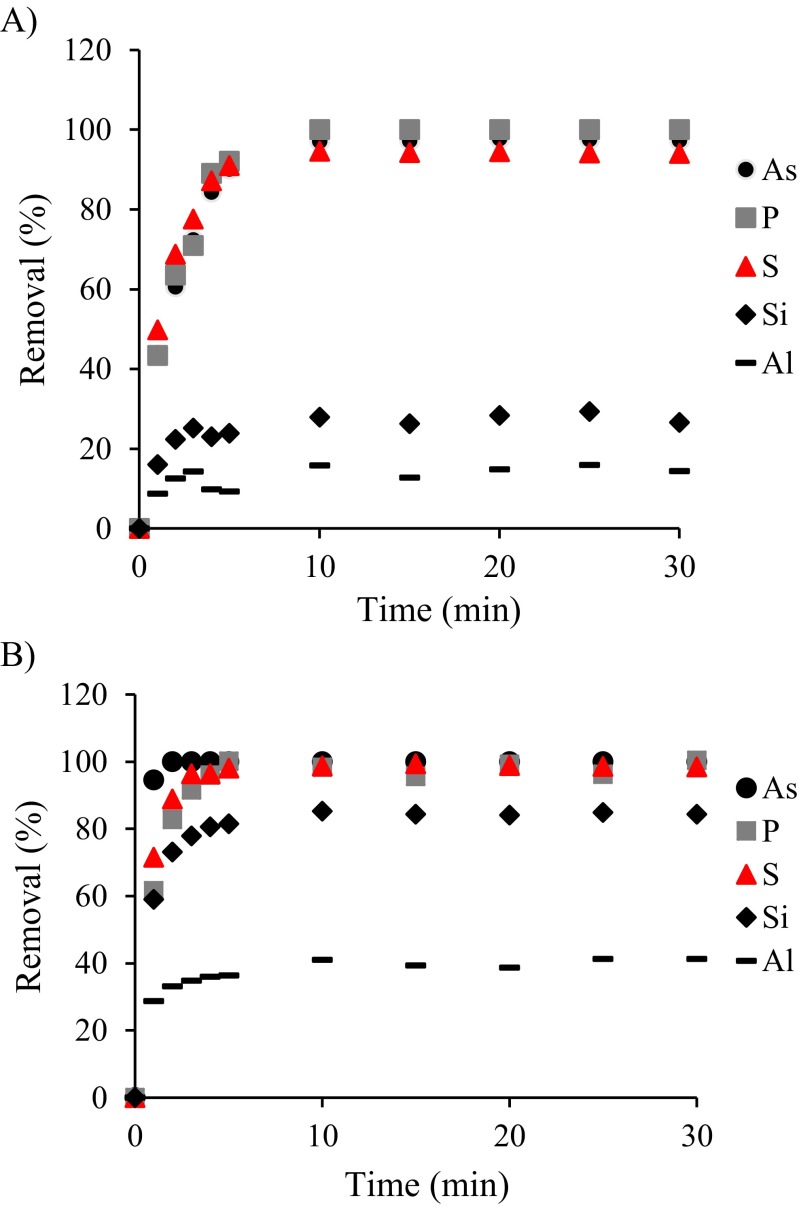
Removal percentages of other elements in the batch tests with **a** operating BDRA neat residue leachate (pH 13) and **b** post-closure effluent and runoff (pH 11.5)

Bauxite residue leachates are enriched in a range of oxyanions that could compete with V on the ion exchange resins. Figure 3 shows the removal percentages of other elements also present in the bauxite residue leachates tested in the batch trials. In the operating BDRA neat residue leachate (pH 13), 100 % of P is removed, followed by 97.8 % of As, 94.6 % of S, 29.4 % of Si and 15.9 % of Al. This means that 1 g of resin removed 15.8 mg Al, 3.6 mg Si, 2.0 mg As, 0.4 mg P and 17.1 mg S. In the post-closure effluent and runoff (pH 11.5), similar results were obtained with higher removal percentages for S, Si and Al (100 % of P and As are removed, followed by 98.5 % of S, 84.9 % of Si and 41.3 % of Al). As the initial concentrations were lower at this pH, in this leachate 1 g of resin removed 1.7 mg Al, 10.2 mg Si, 0.05 mg As, 1.0 mg P and 3.1 mg S.

### Column experiments

**Fig. 4:**
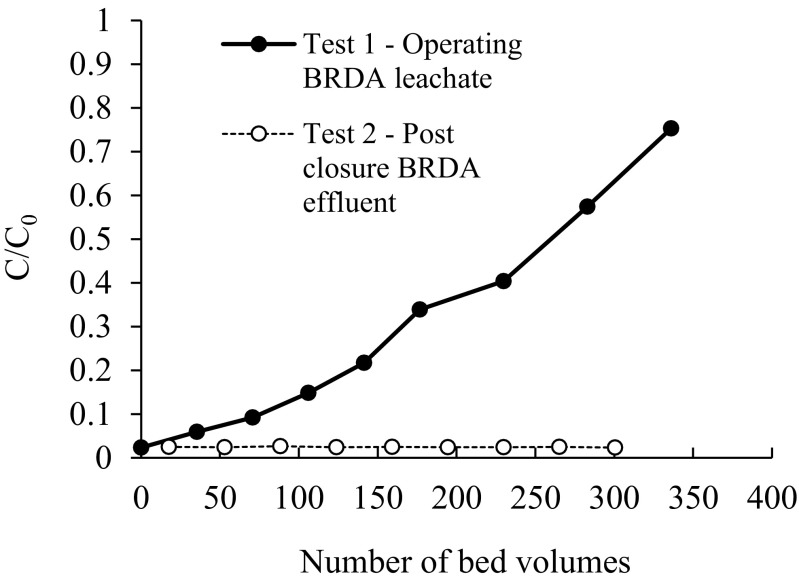
Breakthrough of vanadium with the operating BRDA neat residue leachate (test 1) and the post-closure effluent (test 2)

Figure 4 presents the breakthrough curves in the column experiments with the operating BRDA neat residue leachate (test 1, pH 13) and the post-closure effluent and runoff (test 2, pH 11.5). The breakthrough capacity (BTC) of a column is defined as the resin loading at the point when the effluent concentration is 10 % of C_0_ (Tavakoli et al. 2013). Complete “breakthrough” occurs when the column is exhausted, and the effluent concentration is equal to the influent concentration. In test 1, the breakthrough capacity was reached at 71 bed volumes (400 mL feed solution), and 75 % breakthrough was reached after ~330 bed volumes. In test 2, no breakthrough occurs after 300 bed volumes (Fig. 4). In both tests, no complete breakthrough was reached.

**Fig. 5:**
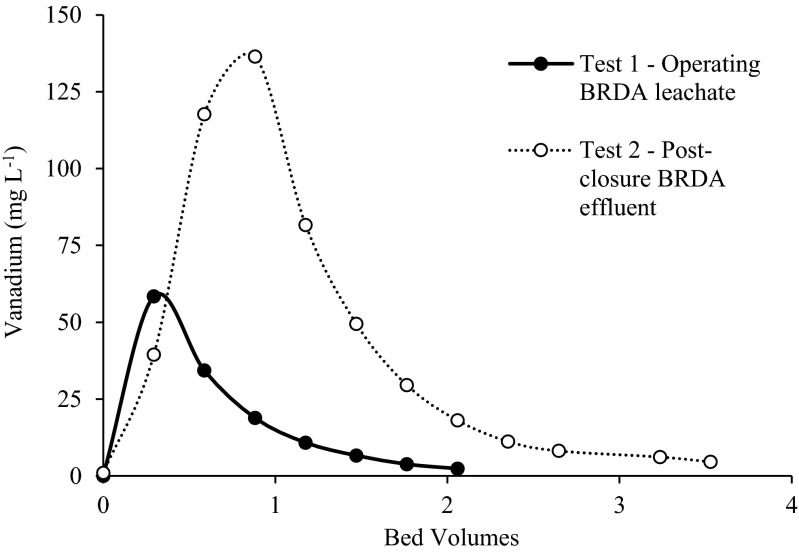
Elution curves of V from the column with 2.0 M NaOH in test 1 and test 2, with pH 13 and 11.5, respectively

The V recovery when the columns were eluted with 2.0 M NaOH is shown in Fig. 5. In test 1, 42 % of vanadium was recovered with 2 bed volumes of NaOH, whereas in test 2 the V recovery was 76 % with 3.5 bed volumes. This value is consistent with others in the literature, that report an 82 % V desorption using 2 M NaOH (Hua et al. 2010).

**Fig. 6:**
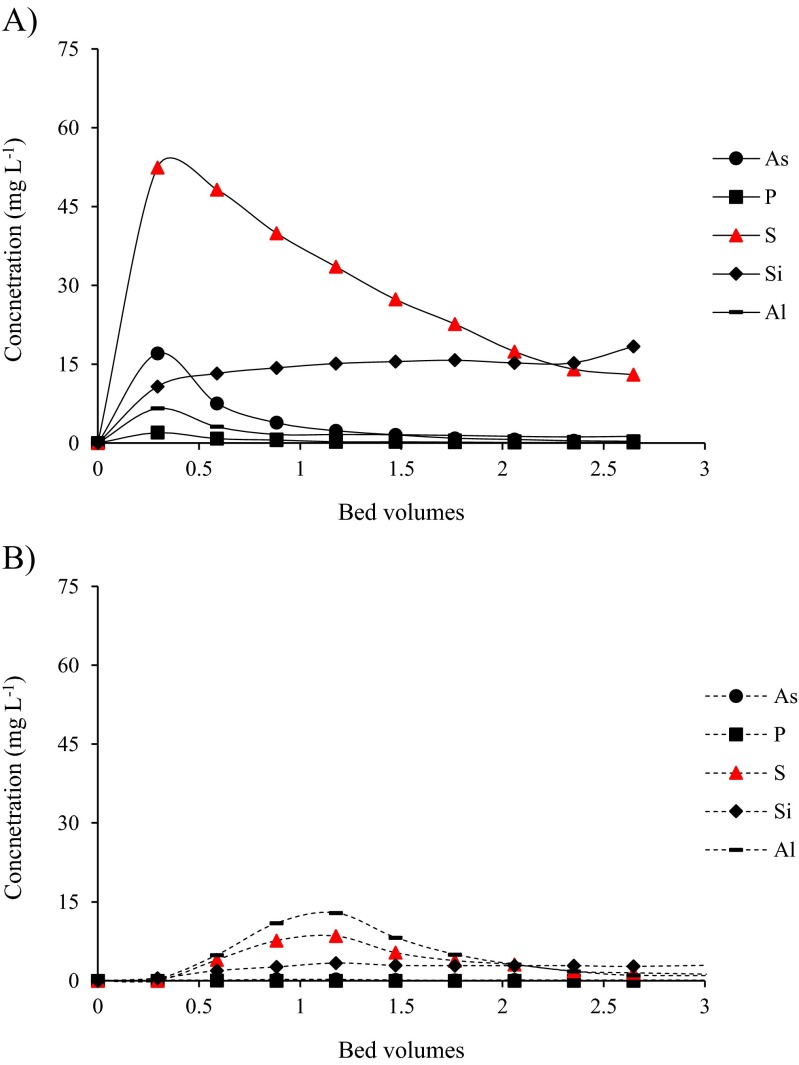
Elution curves of competing elements from the column with 2.0 M NaOH in **a** test 1 with operating BDRA neat residue leachate (pH 13) and **b** test 2 with post-closure effluent (pH 11.5)

Competing elements were also eluted with the NaOH (Fig. 6), especially in test 1 with the operating BRDA leachate (pH 13), where the concentrations of S were similar to vanadium. In test 1, most of the competing ions are eluted in the first 0.5 bed volumes, except S and Si, which has an almost constant concentration. The recoveries were 33 % of S, 28 % of As, 26 % of Si, 18 % of P and 0.2 % of Al. In test 2, the concentrations in the eluate are four times lower, and the peak in the elution curve is obtained at 1.2 bed volumes. In this test, Al is the competing element with higher concentrations, and 86 % is recovered.

### Hydrochemical modelling

Table 6 presents the dominant V species under the tested conditions in the batch trials with the operating BRDA leachate and the post-closure effluent and shows that, in both, the dominant V solution species are hydrogen vanadate (HVO_4_^2−^) and orthovanadate (VO_4_^3−^). At pH 13, divanadate (V_2_O_7_^4−^) is also important, while at pH 11.5, dihydrogen vanadate (H_2_VO_4_^−^) is the third dominant species.

**Table 6:** Dominant ionic species, concentrations (inside brackets, mg L^−1^) in the bauxite residue leachate at pH 11.5 and 13 modelled in PHREEQC using the MINTEQ database with the average concentrations (Table 1)

Element	Operating BRDA neat residue leachate (pH 13)	Post-closure effluent and runoff (pH 11.5)
	Total V = 4.6 mg L^−1^	Total V = 5.3 mg L^−1^
V	HVO_4_^2−^ (7.3)	HVO_4_^2−^ (12.0)
	VO_4_^3−^ (3.1)	VO_4_^3−^ (0.03)
	V_2_O_7_^4−^ (6.7)	H_2_VO_4_^−^ (0.01)
As	AsO_4_^3−^ (2.0)	AsO_4_^3−^ (0.03)
	HAsO_4_^2−^ (0.007)	HAsO_4_^2−^ (0.02)
	H_2_AsO_4_^−^ (1.7 × 10^−9^)	H_2_AsO_4_^−^ (4.8 × 10^−7^)
P	CaPO_4_^−^ (0.7)	HPO_4_^2−^ (1.1)
	PO_4_^3−^ (0.4)	CaPO_4_^−^ (1.2)
	HPO_4_^2−^ (0.02)	PO_4_^3−^ (0.2)
S	SO_4_^2−^ (18.3)	SO_4_^2−^ (1.5)
	NaSO_4_^−^ (1.9)	NaSO_4_^−^ (0.1)
	KSO_4_^−^ (0.02)	KSO_4_^−^ (8.9× 10^−4^)
Si	H_2_SiO_4_^2−^ (2.3)	H_3_SiO_4_^−^ (7.9)
	H_3_SiO_4_^−^ (1.5)	H_2_SiO_4_^2−^ (0.2)
	H_4_SiO_4_ (0.001)	H_4_SiO_4_ (0.2)
Al	Al(OH)_4_^−^ (243.2)	Al(OH)_4_^−^ (7.0)
	Al(OH)_3_ (9.7 × 10^−6^)	Al(OH)_3_ (1.3× 10^−5^)
	Al(OH)_2_^+^(3.8 × 10^−12^)	Al(OH)_2_^+^ (1.8 × 10^−10^)

## Discussion

The primary objective of the study was to test the efficacy of an anion exchange resin for the removal and recovery of vanadium from synthetic bauxite residue leachate solutions. The results show that the resin can be suitable for the removal of V at alkaline pH (11.5 and 13), and the rate at which the resin sorbs V can be predicted by a pseudo-first-order rate equation. Comparing the two feed solutions tested, the post-closure effluent showed a removal rate double that of the operating BRDA leachate and reached higher removal percentages (99.9 vs. 94.3 %). The Freundlich adsorption model had the best fit to the data, indicating that adsorption is non-ideal, and the value of 1/n means a favourable V removal. The Freundlich adsorption capacity constant *K*_f_ was almost double for pH 11.5 compared to pH 13, indicating that V is more rapidly sorbed at lower pH. Two of the predominant V species at pH 11.5 (HVO_4_^2−^ and H_2_VO_4_^−^) have lower charge density than VO_4_^3−^ and V_2_O_7_^4−^ (predominant at pH 13), and therefore, have a higher propensity to interact with the resin surfaces. Hence, sorption is enhanced. Contact time is a critical operational design parameter for water treatment technologies (PIRAMID Consortium 2003) and the short contact time required here shows promise for upscaling and practical application of ion exchange resins for V removal from bauxite residue leachates.

Results from the column tests show that the resin Amberlite® IRA-400 has 20 times less capacity with the simulated operating BDRA leachate (pH 13) than with the simulated post-closure effluent (pH 11.5). At pH 13, competing metal oxyanions, and Al and Si are in high concentrations, as well as the high concentration of OH^−^ of the bauxite residue leachate (0.134 M in the operating BDRA leachate and 4 mM in the post-closure effluent), are likely to explain this lower efficacy. For optimal performance of the ion exchange resin, vanadium must bind more strongly to the resin than the displaced OH^−^ and the competing ions present in the leachate. Published data suggests Amberlite® IRA-400 shows affinity for AsO_4_^3−^>PO_4_^3−^>SO_4_^2−^>Cl^−^>OH^−^ (Korkisch 1989; Tang et al. 2013). The results presented here (Fig. 3) are consistent with the resin affinity and show that simultaneously with hydrogen vanadates, other anions are being removed, which affects the resin capacity.

The ion exchange processes (exchange and regeneration) can be explained by the following equations, where ROH represents the resin in the hydroxide form:

4 $$ HV{O_4}^{2-}+2ROH\ \underleftarrow{{\scriptscriptstyle \to }}{R}_2{HVO}_4+2{OH}^{-} $$

5 $$ {R}_2{HVO}_4+2\kern0.2em {OH}^{-}\underleftarrow{{\scriptscriptstyle \to }}V{O_4}^{3-}+2ROH+{\mathrm{H}}_2\mathrm{O} $$

In the removal of vanadium, hydrogen vanadate (HVO_4_^2−^), the predominant form of V in the bauxite residue leachates, replaces the OH^−^ attached to the exchanging sites of the resin (Eq. 4). All the competing ions identified (AsO_4_^3−^, PO_4_^3−^ and SO_4_^2−^) behave similarly, reducing the resin capacity by occupying the exchange sites. The resin regeneration involves the elution of the resin with a strongly basic solution, in this case, aqueous sodium hydroxide (NaOH 2 M). During regeneration, the trapped negative ions are flushed out and replaced by OH^−^, renewing the exchange capacity of the resin (Eq. 5).

Hydrochemical modelling in PHREEQC indicates some mineral precipitation is possible in these solutions, and that many of these precipitates include competing ions. Key solid phases are Ba_3_(AsO_4_)_2_ (saturation index SI 11.38); Ca_3_(PO_4_)_2_ (SI 0.59); Ca_5_(PO_4_)3OH (SI 11.92); Mg_3_Si_2_O_5_(OH)_4_ (SI 5.85); AlOOH (SI 0.28) and Mg_2_Si_3_O_7_.5OH:3H_2_O (SI 0.29). However, no visual evidence of precipitation was noted, and the removal of these phases from solution and their presence in the eluate in the batch and the column tests results suggests that the competing ions are exchanged in the resin. The presence of the competing elements in the eluate (Fig. 6) also points the need for more focused research to separate and purify vanadium for further recovery.

Even with the lower ionic strength influent of the post-closure effluent, there is always a fraction of V (2–3 %) that is not retained in the resins (Fig. 4). There are two possible explanations for these results—OH^−^ ions in the leachate are eluting the vanadate ions from the resins, or the bed volume is insufficient for the removal and recovery of V.

Management of post-closure BRDA effluents is an increasingly prominent issue for the alumina industry (Clune et al. 2015; Higgins et al. 2015). The Ajka (Hungary) spill in 2010 highlighted some of the risks associated with BRDA leachates (Mayes et al. 2016), and there is increased R&D efforts to manage these issues given the potential longevity of alkaline leachate generation and metal(loid) enrichment (Buckley et al. 2016). The use of ion exchange resins to strip out elements that are problem pollutants and critical resources simultaneously would complement passive pH management efforts well. With the case of vanadium, this is particularly important given it appears to be only partially removed through precipitation from bauxite residue leachates during buffering to *circum*-neutral pH, contrary to Al and As (Burke et al. 2013). Further work is required to establish the viability of the resin for V recovery from bauxite residue leachate under environmental conditions and to test with higher bed volumes and configurations that could effectively remove competing ions (e.g. sequential removal using differing resins and multiple columns).

## Conclusions

This study has shown that anion exchange resins can be used for metal removal and recovery from bauxite residue leachates in a highly alkaline pH range (up to 13). The results showed that using simulated undiluted bauxite residue leachate as feed solution limited the resin efficacy, due to the presence of competing ions, notably OH^−^. However, the resins are very effective at V removal for simulated post-closure bauxite residue disposal areas (BRDA) effluent, which has a lower ionic strength. Column experiments demonstrate that V is readily eluted from the resins in concentrations similar to some industrial process liquors which holds promise for recovery and recycling of V into downstream industrial processes. Further research is required to scale-up laboratory findings. This should include assessment pretreatments and optimisation of operating parameters, such as flow rate and bed height. This will help facilitate life cycle assessments of anion exchange resins as a potentially efficient and cost-effective option for both the treatment of bauxite residue leachates and the recovery of metals of critical importance such as vanadium.
